# Prevalence and genotype distribution of human papillomavirus infections in Beijing, China between 2016 and 2020

**DOI:** 10.1186/s12985-023-01959-7

**Published:** 2023-01-18

**Authors:** Wei Zhang, Nan Guo, Baoping Li, E Shang, Jinxia Wang, Mei Zhang, Ximing Yang

**Affiliations:** Department of Clinical Laboratory, Dongzhimen Hospital, Beijing University of Chinese Medicine, No. 5, Haiyuncang, Dongcheng District, Beijing, 100700 China

**Keywords:** Human papillomavirus, Prevalence, Genotype, Cervical cancer, Vaccine

## Abstract

**Background:**

Certain types of human papillomavirus (HPV) induce long-lasting infections that cause cervical cancer. This study evaluated the prevalence of HPV infections and the distribution of their genotypes among clinic patients and healthy women in Beijing, China.

**Methods:**

Cervical specimens were collected from 12,100 patients and 1176 subjects who underwent physical examinations at Dongzhimen Hospital, Beijing University of Chinese Medicine, between March 2016 and September 2020. HPV genotyping was performed using commercial kits designed to detect 15 high-risk and 2 low-risk HPV genotypes.

**Results:**

There was a higher overall prevalence of HPV among the clinic patients (21.0%) than among the healthy women (11.9%). The most common HPV genotypes among the patients were: HPV-52 (5.4%), HPV-16 (3.4%), HPV-58 (3.2%), HPV-51 (2.6%), HPV-39 (2.0%), HPV-56 (2.0%), and HPV-66 (2.0%). Among the healthy women: HPV-52 (3.0%), HPV-51 (1.8%), HPV-58 (1.6%), HPV-66 (1.5%), HPV-16 (1.2%), HPV-56 (1.2%), and HPV-18 (1.1%). Multiple HPVs were detected in 29.1% of the gynecological outpatients and in 23.6% of the women receiving physical examinations. The most common pairs of HPV types detected were HPV-52 and HPV-16 in the clinic patients, and HPV-52 and HPV-56 in the healthy women. Age-specific HPV positivity and peak prevalence were observed among the individuals in both groups for women aged less than 25 years and those between 61 and 65 years of age.

**Conclusions:**

Our results provide current estimates of HPV prevalence and genotypes in the Beijing region. The epidemiological characteristics observed also provide a reference for the development of cervical cancer screening strategies and selection of HPV vaccine antigen targets for this region. A comparison of these HPV prevalence data with those from other regions suggests that regional vaccines may be an important direction for future research.

## Introduction

Cervical cancer (CC) is a highly preventable disease, yet remains the fourth most frequently diagnosed cancer, and fourth leading cause of cancer-related mortality, among women worldwide [1]. In 2020, it was reported that 604,000 women were diagnosed with CC worldwide, and approximately 342,000 women died from the disease [1]. In May 2018, the World Health Organization called for a global initiative to eliminate CC as a public health problem [2]. However, since 2000, the incidence and mortality rates of CC in China have continued to increase significantly [3, 4]. It is estimated that 111,820 new cases and 61,579 deaths have already been attributed to CC in 2022 [3].

Persistent infection with human papillomavirus (HPV) is the main etiologic factor for CC [5]. There are more than 200 genotypes of HPV which belong to 49 species and five genera [6]. The International Agency for Research on Cancer has classified twelve HPV types (16, 18, 31, 33, 35, 39, 45, 51, 52, 56, 58, and 59) which are referred to as Group 1, as carcinogenic agents with sufficient evidence in humans. Meanwhile, types 26, 53, 66, 67, 68, 70, 73, and 82 are recognized as agents with limited evidence in humans [7]. HPV-16 and HPV-18 represent the most oncogenic types, and they are responsible for approximately 70% of CC cases worldwide [8]. When HPV types 31/33/45/52/58 are additionally considered, this percentage increases to 90% [9].

Over the last few decades, trends in CC incidence and mortality have been observed to vary in different countries [5]. Similarly, the prevalence and genotype distribution of HPV has been observed to vary among countries worldwide [10–12]. The latter is especially relevant in China, with its vast regions encompassing large and multiracial populations [13]. Research regarding the prevalence and genotype distributions of HPV in different areas is critical for CC screening efforts and for evaluations of HPV vaccine efficacy among females in China. Therefore, we conducted a comprehensive analysis of HPV prevalence characteristics among both female clinical patients and healthy women receiving physical examinations in Beijing, China.

## Materials and methods

### Study population

For this cross-sectional and retrospective study, a total of 13,276 patients were enrolled from Dongzhimen Hospital Beijing University of Chinese Medicine (Beijing, China) between March 2016 and September 2020. These patients visited the hospital for various reasons, including: leukorrhagia, menstrual disorders, pelvic inflammation, vaginitis, cervicitis, undiagnosed abdominal pain, genital warts, cervical intraepithelial neoplasia, and for physical examination. This study was approved by the Ethics Committee of the Dongzhimen Hospital Beijing University of Chinese Medicine (Grant No.: 2022DZMEC-094-01).

### Sample collection, HPV detection, and genotyping

All of the subjects were asked to refrain from sexual activity and avoid washing their genital area for 48 h prior to sample collection. Cervical samples were obtained by using a cervical brush and then were placed in a preservative buffer solution. The samples were stored at 2–8 °C for no more than 7 days. DNA was isolated using a nucleic acid extraction reagent (Shanghai ZJ Bio-Tech Co., Ltd., Shanghai, China) in compliance with the manufacturer’s instructions.

For HPV detection and genotyping, two commercial HPV Genotyping Kits (Shanghai ZJ Bio-Tech Co., Ltd.) were used to detect fifteen high risk (HR)-HPV types (16, 18, 31, 33, 35, 39, 45, 51, 52, 56, 58, 59, 66, 68, and 82) and a combination of two low-risk (LR)-HPV types (6/11) with use of TaqMan real-time fluorescent quantitative polymerase chain reaction (PCR) using a Slan-96P Real-time PCR System assay (Hongshi Medical Technology Co., Ltd., Shanghai, China). Samples were reported to be HPV positive if any of the 17 HPV DNA types were detected. In brief, each 4-μL sample of DNA was mixed with 35.6 μL of PCR Mix and 0.4 μL DNA Taq polymerase. Amplification of HR-HPV types included the following steps: 94 °C for 2 min, followed by 40 amplification cycles (denaturation at 93 °C for 10 s, annealing and elongation at 62 °C for 31 s). Single-point fluorescence was detected at 62 °C. To amplify LR-HPV types, the following steps were programmed: 37 °C for 2 min, 94 °C for 2 min, and a total of 40 amplification cycles (denaturation at 93 °C for 15 s, annealing and elongation at 60 °C for 60 s). Single-point fluorescence was detected at 60 °C. The detection limits of the assays were 1 × 10^4^ copies/mL and 1 × 10^3^ copies/mL, respectively. To quantify the amount of HPV in the samples and to avoid false-negative test results, internal quality control was performed alongside the samples. Quality controls were included in all of the experiments, including DNA amplification and genotyping, with positive and negative controls included in the PCR assays.

### Statistical analysis

All of the statistical analyses performed for this study used SPSS 21.0 for Windows. Prevalence of HPV infection, genotype distribution, and both single and multiple HPV infections were analyzed individually. Prevalence of HPV infections with single- versus multiple-HPV genotypes in different groups were compared using the Chi-square test. Binomial distribution analysis was used to calculate 95% confidence interval (95%CI). Factors associated with HPV prevalence were evaluated and reported as odds ratios (ORs) and 95% CI. Two-sided *P* values less than 0.05 were considered statistically significant. The test criterion was *α* = 0.05 and was used throughout the study. If one or more of the expected numbers was ≤ 5, or the *P *value was close to 0.05, Fisher’s exact test was applied.

## Results

### Study subjects

A total of 13,276 subjects were included in this study with a median age of 39 years (range 18–85). Among these patients, 12,100 experienced various gynecopathy conditions, including: leukorrhagia, menstrual disorders, pelvic inflammation, vaginitis, cervicitis, undiagnosed abdominal pain, genital warts, or cervical intraepithelial neoplasia. In addition, 1176 healthy women were included who underwent physical examinations.

### Specific prevalence of HPV types

**Fig. 1:**
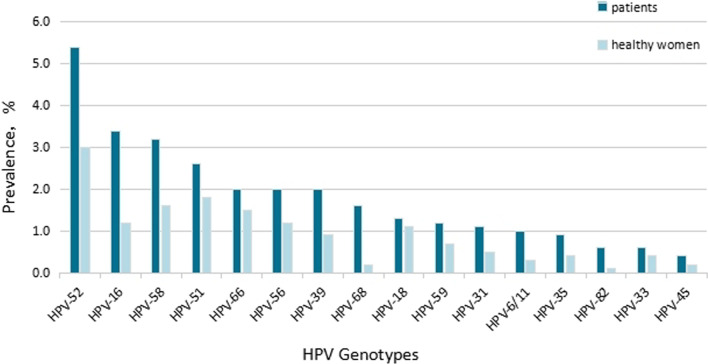
Prevalence of various HPV genotypes detected among the patients and healthy women examined

A total of 2675 subjects (20.1%, 95%CI 19.4–20.8) were positive for HPV DNA. According to group, the prevalence of HPV DNA was 21.0% (95%CI 20.2–21.7) among the clinic patients and 11.9% (95%CI 10.0–13.9) among the healthy controls (Table 1, Fig. 1). Furthermore, the positive rate of HR-HPV was 20.5% (95%CI 19.8–21.2) and 11.7% (95%CI 9.8–13.6) for the two groups, respectively. There were seven HPV types that were most commonly detected: HPV-52 (5.4%, 95%CI 5.0–5.8), HPV-16 (3.4%, 95%CI 3.1–3.8), HPV-58 (3.2%, 95%CI 2.9–3.5), HPV-51 (2.6%, 95%CI 2.3–2.9), HPV-39 (2.0%, 95%CI 1.8–2.3), HPV-56 (2.0%, 95%CI 1.8–2.3), and HPV-66 (2.0%, 95%CI 1.7–2.2). These were detected in patients infected with single or multiple HPV types. Among the healthy women, HPV-52 (3.0%, 95%CI 2.0–3.9), HPV-51 (1.8%, 95%CI 1.1–2.6), HPV-58 (1.6%, 95%CI 0.9–2.5), HPV-66 (1.5%, 95%CI 0.9–2.3), HPV-16 (1.2%, 95%CI 0.6–1.9), HPV-56 (1.2%, 95%CI 0.6–1.9), and HPV-18 (1.1%, 95%CI 0.6–1.8) were most often detected. Between the two groups, there were significant differences (*P* < 0.05) between the prevalences of: HPV-52 (*P* < 0.05, OR: 1.863, 95%CI 1.319–2.631), HPV-16 (*P* < 0.05, OR: 2.963, 95%CI 1.733–5.063), HPV-58 (*P* < 0.05, OR: 2.033, 95%CI: 1.278–3.235), HPV-39 (*P* < 0.05, OR: 2.207, 95%CI: 1.203–4.049), HPV-6/11 (*P* < 0.05, OR: 3.058, 95%CI 1.128–8.292), HPV-68 (*P* < 0.05, OR: 9.314, 95%CI 2.310–37.565), and HPV-82 (*P* < 0.05, OR: 6.837, 95%CI 0.949–49.263). The prevalence of certain HPV types (e.g., 18, 31, 33, 35, 45, 51, 56, 59, and 66) was higher among patients than among the controls, although these differences were not statistically significant. The pooled OR for the patients with mixed gynecopathy associated with the presence of any HPV type was 1.961 (95%CI 1.635–2.352).

**Table 1:** Human Papillomavirus (HPV) types in 12,100 patients and in1176 healthy women with single or multiple types

HPV subtypes	Patients		Healthy women		χ^2^	*P *value
	No	Frequency for all patients (%) (95%CI)	No	Frequency for all healthy women (%) (95%CI)		
Any HPV	2535	21.0 (20.2–21.7)	140	11.9 (10.0–13.9)	54.509	< 0.001
HR-HPV	2477	20.5 (19.8–21.2)	138	11.7 (9.8–13.6)	51.719	< 0.001
52	654	5.4 (5.0–5.8)	35	3.0 (2.0–3.9)	12.850	< 0.001
16	417	3.4 (3.1–3.8)	14	1.2 (0.6–1.9)	17.364	< 0.001
58	391	3.2 (2.9–3.5)	19	1.6 (0.9–2.5)	9.349	0.002
51	314	2.6 (2.3–2.9)	21	1.8 (1.1–2.6)	2.854	0.91
39	247	2.0 (1.8–2.3)	11	0.9 (0.4–1.5)	6.880	0.010
56	244	2.0 (1.8–2.3)	14	1.2 (0.6–1.9)	3.838	0.050
66	241	2.0 (1.7–2.2)	18	1.5 (0.9–2.3)	1.191	0.320
68	189	1.6 (1.3–1.8)	2	0.2 (0.0–0.4)	14.645	< 0.001
18	161	1.3 (1.1–1.5)	13	1.1 (0.6–1.8)	0.420	0.583
59	151	1.2 (1.1–1.5)	8	0.7 (0.3–1.2)	2.919	0.092
31	137	1.1 (1.0–1.3)	6	0.5 (0.2–1.9)	3.892	0.053
6/11	125	1.0 (0.9–1.2)	4	0.3 (0.0–0.7)	5.348	0.021
35	107	0.9 (0.7–1.0)	5	0.4 (0.1–0.9)	2.701	0.130
33	78	0.6 (0.4–0.7)	5	0.1 (0.0–0.3)	0.831	0.442
82	70	0.6 (0.5–0.8)	1	0.4 (0.1–0.9)	4.907	0.027
45	49	0.4 (0.3–0.5)	2	0.2 (0.0–0.4)	0.992	0.319

### Distribution of HPV infections

There were a total of 2535 patients that were positive for HPV DNA (Table 2). Of these, 70.9% (95%CI 69.1–72.6) were infected with a single HPV type; 20.4% (95%CI 18.9–21.9) were infected with two HPV types; 6.5% (95%CI 5.5–7.5) were infected with three HPV types; 1.4% (95%CI 1.0–1.9) were infected with four HPV types; 0.6% (95%CI 0.3–0.9) were infected with five HPV types; and 0.2% (95%CI 0.0–0.4) were infected with six types of HPV. Among the healthy women, 140 were positive for HPV DNA. Of these, 76.4% (95%CI 69.3–82.9) were infected with a single HPV type; 20.7% (95%CI 14.3–27.1) were infected with two HPV types; 2.1% (95%CI 0.0–5.0) were infected with three HPV types; and 0.7% (95%CI 0.0–2.1) were infected with four types of HPV. There were no significant differences between the two groups regarding their HPV profiles.

**Table 2:** Human Papillomavirus (HPV) types in 2535 infected patients and in 140 infected healthy women with single or multiple types

HPV types	Patients		Healthy women	
	No. positive	% for all infected patients (95%CI)	No. positive	% for all infected healthy women (95%CI)
Single infections	1797	70.9 (69.1–72.6)	107	76.4 (69.3–82.9)
Double infections	517	20.4 (18.9–21.9)	29	20.7 (14.3–27.1)
Triple infections	165	6.5 (5.5–7.5)	3	2.1 (0.0–5.0)
Quadruple infections	36	1.4 (1.0–1.9)	1	0.7 (0.0–2.1)
Quintuple infections	15	0.6 (0.3–0.9)	NA	NA
Sextuple infections	5	0.2 (0.0–0.4)	NA	NA
Total no. of subjects with multiple infections	738	29.1 (27.4–30.8)	33	23.6 (17.1–30.7)

The most commonly detected double HPV types are listed in Table 3. HPV-52, HPV-51, HPV-58, and HPV-16 combined with another HPV type accounted for at least 50% of the total number of clinic patients with double infections. Meanwhile, among the healthy women, HPV-52 and HPV-56, HPV-52 and HPV-59, HPV-52 and HPV-66, and HPV-51 and HPV-66 were the common double infections detected.

**Table 3:** Human Papillomavirus (HPV) types detected in 2535 infected patients and in 140 infected healthy women with double infections

HPV types	Patients		Healthy women	
	No. positive	% for all infected patients	No. positive	% for all infected healthy women
52 and 16	37	1.5	1	0.7
52 and 58	22	0.9	0	0.0
52 and 66	19	0.7	2	1.4
52 and 39	18	0.7	1	0.7
52 and 51	17	0.7	0	0.0
58 and 68	17	0.7	0	0.0
16 and 58	16	0.6	0	0.0
52 and 56	12	0.5	3	2.1
52 and 59	10	0.4	2	1.4
16 and 51	10	0.4	1	0.7
58 and 39	9	0.4	0	0.0
51 and 56	9	0.4	0	0.0
51 and 66	9	0.4	2	1.4
16 and 39	8	0.3	1	0.7
58 and 56	8	0.3	0	0.0
52 and 18	7	0.3	0	0.0
16 and 56	7	0.3	0	0.0
52 and 68	6	0.2	0	0.0
58 and 51	7	0.3	0	0.0
16 and 18	6	0.2	0	0.0
Other double infections	263	10.2	16	11.6
Total no. of women with double infections	517	20.4	29	20.7

### Age-specific prevalence of HPV infection

**Fig. 2:**
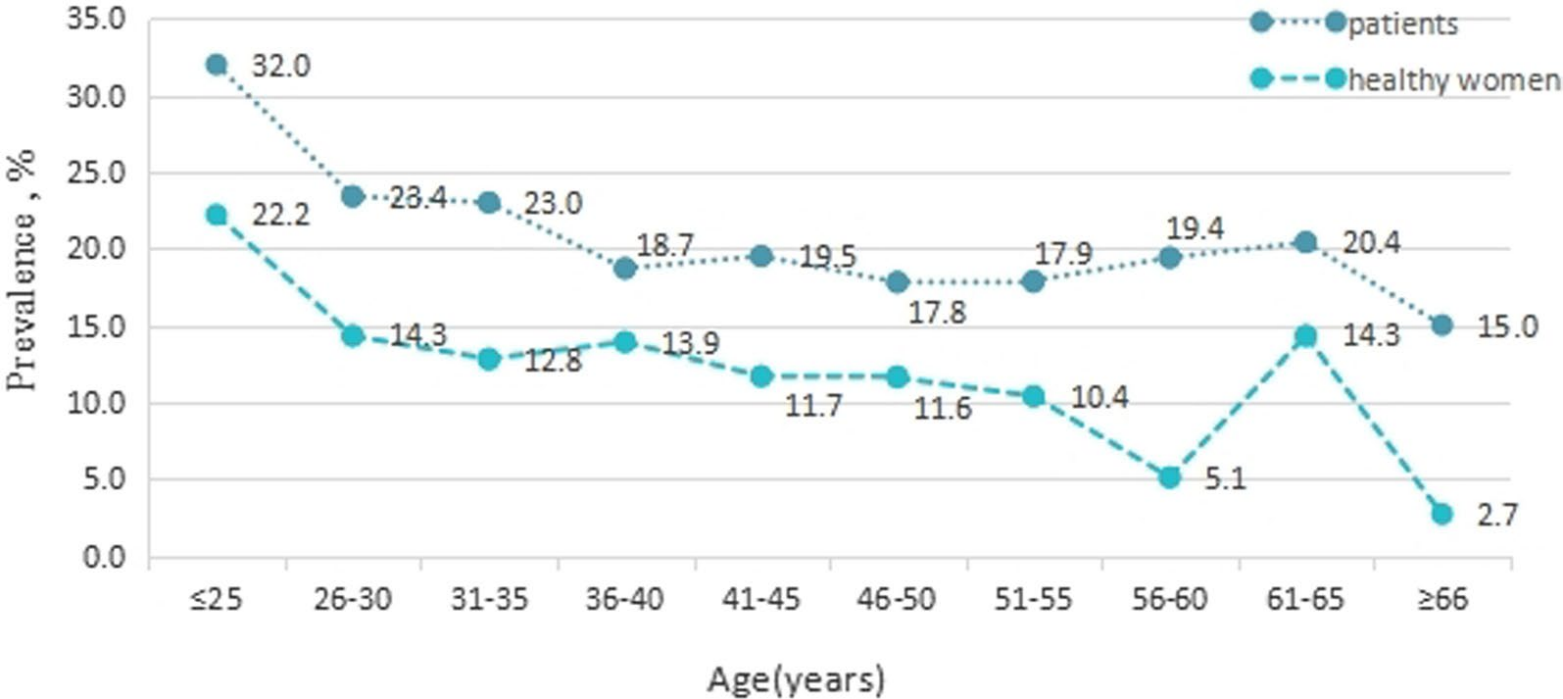
Prevalence of HPV types tested among ten age groups of our cohort. To evaluate possible trends in HPV prevalence with age, the cases examined were stratified into ten groups according to age. HPV infections detected in both groups exhibited a bimodal distribution with age, with two peaks in prevalence observed for the ≤ 25 years and 61–65 years groups

To explore whether the prevalence of HPV exhibits an age-dependent trend in our cohort, the cases were stratified into ten groups according to age (Fig. 2). In both the clinical patient and healthy women groups, the highest overall prevalence of HPV infections was observed among the subjects that were ≤ 25 years of age. Moreover, the prevalence of HPV infection was significantly higher among the patients than among the healthy women for this age group (32% vs. 22.2%, respectively; *P* < 0.05). The 26–30 years age group showed a marked decrease in HPV prevalence for both groups (23.4% and 14.3%, respectively) that was followed by a gradual decrease in prevalence in both groups. For the patient group, this included a decrease to 17.8% in the 46–50 years group, an increase to 20.4% in the 61–65 years group, and then a decrease to 15.0% in the ≥ 66 years group. Meanwhile, in the healthy women group, a gradual decrease led to a low point at 5.1% for the 56–60 years group, followed by a second smaller peak in HPV prevalence (14.3%) in the 61–65 years age group, and then 2.7% for the ≥ 66 years group.

## Discussion

This cross-sectional study reports the prevalence of HPV infections and genotype distributions among two groups of women, clinic patients and healthy women, between 2016 and 2020 in Beijing, China. These epidemiological data support the availability of HPV vaccines in this region. Previous studies have reported that the prevalence of HPV varies considerably in China, from 6.2 to 50.64% [14, 15]. It is possible that differences in study setting (clinic-based vs. population-based), sampling periods, social economy, and geographical regions contribute to this range. Outpatient samples have been used in most studies of HPV epidemiology [16]. Since outpatients usually come to a hospital to address medical issues, their HPV prevalence may differ from that of healthy women who participate in routine physical examinations [17]. Therefore, in the present study, we examined epidemiological data from both outpatients and healthy women in Beijing. Ultimately, 17 distinct HPV types were detected and the overall prevalence of HPV was determined to be 21.0% among the clinical patients and 11.9% among the healthy women. These results are consistent with those of previous studies which reported HPV prevalence to be 21.06% among a clinic-based female group and 12.18% among a healthy female group in Beijing [18, 19].

Some multicenter studies have shown that geographical variations contribute to the prevalence and distribution of HPV in mainland China [20, 21]. For the present study, we further pooled region-specific data available for China to analyze the prevalence of HPV among the different regions in China between 2008 and 2020 (Table 4). To reduce the impact of the different case sources involved, most of the studies selected for consideration were based on general population data derived from cervical cancer screening programs (CCSs) or from healthy women undergoing a physical examination. Certain case-controlled trials and multicenter trials were also considered. According to the population-based data, the overall prevalence of HPV in Northern China is 8.92% in Shanxi [22], 10.3% in Liaoning [23], 14.5% in Inner Mongolia [24], and 14.71% (Tianjin) [25] in Northern China. In Southern China in the Guangdong [26], Fujian [27], and Guangxi [28] provinces, the rates of HPV prevalence vary from 16.2 to 19.5%. Meanwhile, the regions of Western and Middle China have similar HPV rates of 13.5% (Shannxi) [29], 12.6–15.29% (Sichuan) [30, 31], 12.09% (Henan) [32], 13.56% (Jiangxi) [33], and 13.88% (Hunan) [34]. In contrast, HPV prevalence in Eastern China is reported to vary greatly from 10.6% (Jiangsu) [17] to 22.41% (Zhejiang) [35]. In Zhejiang [36], data from routine physical examinations performed indicate the prevalence of HPV is 13.6%. It remains unclear why different studies of the same area (Hangzhou) that were collected during the same periods (2016–2020) show such large differences in HPV prevalence. A possible reason is that the former study detected 29 HPV types, whereas the latter study detected 25 HPV types. It remains for a large sample to be analyzed in order to verify whether the large differences in rates reported are due to different targeted HPV genotypes or populations. A population-based multicenter study showed that the prevalence of HR-HPV infections varied from 6.3 to 21.55% in China [20, 21, 37]. Considering the available data, we predict the mean prevalence of HPV in the overall population of China ranges from 10 to 20%.

**Table 4:**
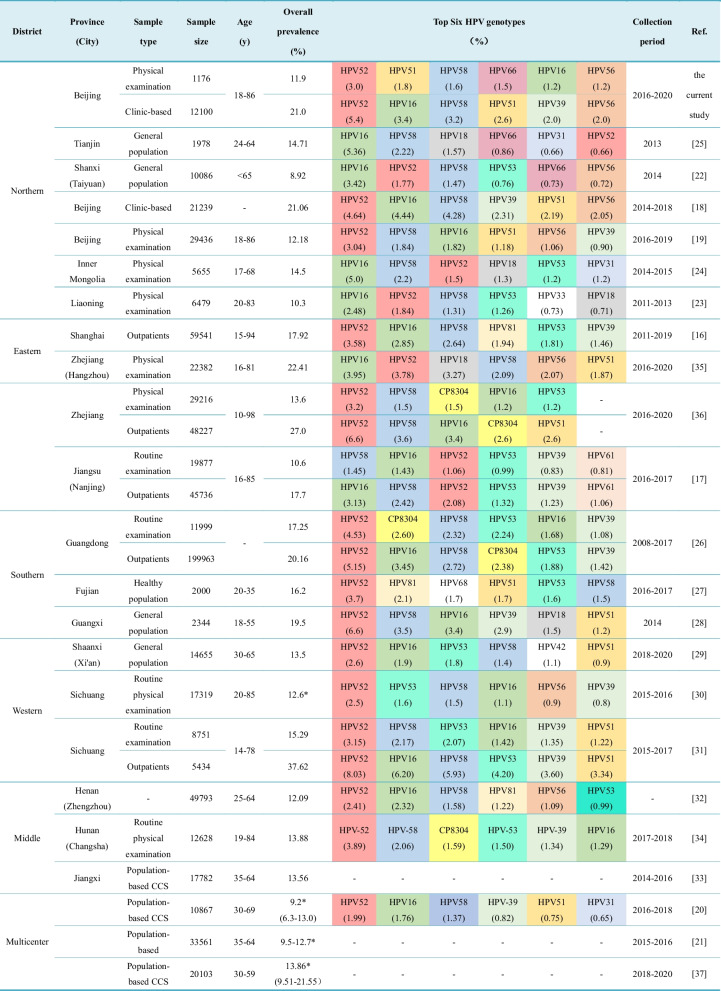
Prevalence of HPV genotypes in China

– There were no original data available from the references

^*^HR-HPV prevalence (%)

Compared with studies based on general populations, the prevalence of HPV among clinic-based patients varies greatly [38]. For example, the prevalence rates of HPV among clinic patients include 17.92% in Shanghai [16], 22.82% in Luoyang (Henan province) [39], 34.58% in Taizhou (Jiangsu province) [40], 38.3% in the Fujian province [41], 41.4% in Hangzhou (Zhejiang province) [42], and 50.64% among females in Tianjin [15]. The reason for the large difference in rates is mainly due to complex pathologies of the study subjects who are seen at hospital for various gynecopathies, including cervical intraepithelial neoplasia, genital warts, and other diseases. The present results are consistent with those of previous studies, with the prevalence of HPV in clinic-based patients being higher than in the general population.

Consistent with the results of previous studies [18, 19], we found HPV-52 to be the most prevalent genotype among single and co-infections in the gynecological patients and healthy women examined in this study. HPV-16 is the most carcinogenic HPV type, and it generally accounts for half of cervical cancer cases [43]. In the current study, HPV-16 was ranked second among our gynecological patients and fifth among the healthy women receiving physical examinations. We observed the prevalence of HPV-16 in our patients to be higher among the women undergoing healthy examinations. Persistent HPV-16 infections have been associated with precancerous lesions of cervical cancer [44]. Moreover, HPV-16 is responsible for ~ 85% of all other HPV­related, non­cervical cancers [45]. In the present study, the OR for HPV-16 was 2.963 among the two groups examined, indicating that the prevalence of HPV-16 in the clinic patients was 2.963 times higher than in the healthy women. It is also worth noting that HPV-66 ranked in the top six of the HPV types detected in our study, yet it is not the main HPV type detected in other regions. Similar observations have been reported for Tianjin, Shanxi, and Beijing [19, 22, 25]. Our data suggest that HPV-66 is possibly a local characteristic genotype of Northern China. Except for HPV types 52, 16, and 18, the prevalence of HPV-53 is in the top three in the general population of Western China [e.g., Shaanxi (1.8%) [29], Sichuan (1.6–2.07%) [31]]. These findings further support the observation that distributions of HPV genotypes are characteristic of the geographical diversity in China. For example, HPV types 16, 39, 51, 52, 53, 56, and 58 are the most common types throughout China, while HPV types 18, 31, 33, 45, 66, 81, and CP8304 have been reported in only certain areas of China (Table 4).

It remains unclear whether competitive or cooperative interactions exist among co-infecting HPV genotypes. Infection by multiple HPV types has been associated with a greater risk of CC than infection by a single HPV type [46]. An association between multiple HPV infections and high viral loads with infection persistence was observed in a study conducted in Mexico [12]. In contrast, a group of patients in Korea with multiple HPV infections displayed persistent and longer durations for their HPV infections than patients with single HPV infections [47]. In the present study, multiple HPVs were detected in 29.1% of the gynecological outpatients and in 23.6% of the healthy women receiving physical examinations. In addition, infections involving more than three HPV types were observed among 8.7% of the gynecological outpatients and among 2.9% of the healthy women. There were more than three HPV types that were detected in some patients, but not in any of the 140 healthy women receiving physical examinations. A larger sample size of women positive for multiple HPV genotypes could provide further insight into whether competitive interactions exist among HPVs.

Based on the results of the current study, it is observed that cervical HPV infections exhibit a bimodal distribution with age. A similar overall trend was observed for age-specific HPV positivity and peak prevalence among individuals aged less than 25 years and aged 61–65 years among gynecological patients and healthy females. The first peak corresponds with age at first sexual intercourse. Number of births is also recognized as a risk factor. In contrast, incidence of cervical HPV infections has previously been reported to have similar persistence rates independent of age [17, 26]. A possible reason for this peak may be an extended duration of exposure to risk factors for women engaging in sexual behavior at a younger age and having a greater number of births, the latter leading to more serious cervical injury [22]. It is now accepted that HPV infections may be latent and are controlled by cellular immune surveillance which induces viruses to maintain very low copy numbers in order to escape detection [48]. In the menopausal or aging state, moderate reactivation may occur [48]. These conditions are consistent with the second peak in HPV prevalence that we observed for women aged 61–65 years in the present study.

CC can be effectively controlled with organized screening and vaccination programs. HPV causes CC in three necessary steps: acquisition, persistence linked to development of precancer, and invasion [49]. Vaccination of adolescents and young adults would interrupt the acquisition of HPV and ensure subsequent elimination of the second precancer step [49]. An HPV vaccine was approved by the US Food and Drug Administration in 2006 for women, and in 2009 for men. The vaccine provides protection against about 70% of CC-causing strains of HPV [50]. Since 2016, imported bivalent (targeting HPV-16 and -18), quadrivalent (targeting HPV 16, 18, 6, and 11), 9-valent (targeting HPV types 16, 18, 31, 33, 45, 52, 58, 6, and 11), and domestically produced HPV16/18 recombinant bivalent vaccines (Xiamen Innovax Biotech, Xiamen, China) have been successively approved by Chinese authorities for marketing in the Chinese mainland [51, 52]. However, the HPV vaccination rate only ranges from 2.64 to 11.0% in China, which is much lower than the rates in most other countries [53–55]. There are many reasons for this low vaccination rate. Among female college students, the high cost of the vaccine (57.7%) and concerns regarding adverse events (56.0%) were cited as the main reasons for not receiving an HPV vaccine [51]. Meanwhile, the supply of vaccines is currently insufficient and the vaccine is not included in the national immunization program. The latter may also influence or delay individuals’ willingness to receive HPV vaccination [56].

Regional variations in the distribution of certain HPV types should be taken into account when creating vaccines that are tailored to different geographic regions [46]. The 9vHPV vaccine prevents infection and disease related to HPV types 31, 33, 45, 52, and 58 in susceptible populations, and generates an antibody response to HPV 6, 11, 16, and 18. However, the 9vHPV vaccine does not prevent infection and disease related to HPV types beyond the nine types covered by the vaccine [57]. In the present study, four nonvaccine HR-HPV types (HPV 51, 56, 53, and 39) ranked among the top seven types of HPV detected in persons of China ancestry, yet only a few HR-HPV types were detected in individuals of other ancestries. It has been hypothesized that HPV 51, 56, 53, and 39 may be better adapted to individuals of Chinese ancestry. Additionally, the increased prevalence of these four types may be a consequence of ancient geographic isolation. The present data indicate that the large majority of HR-HPV types identified are encompassed by the currently produced 9-valent vaccine. However, our data also indicate that the addition of HPV types 39, 51, 53, and 56 could be advantageous for populations in China. Further studies are needed to confirm whether these types are frequently detected in cervical cancer cases in China.

There were limitations associated with the present study. First, due to the retrospective design of this study which relied on a medical record system, each observed clinical diagnosis could not be accurately stratified due to its complexity and non-uniformity. Thus, a prospective cohort study is needed to evaluate the relationship between HPV genotype and disease type, especially cervical intraepithelial neoplasia. Second, according to the cross-sectional design of this study, observed associations cannot be interpreted as temporally linked with infection [58]. Third, the numbers of healthy women who received a physical examination and were included in this study were relatively small. However, to our knowledge, this is the first study to present data regarding the prevalence characteristics of HPV in both outpatient and healthy groups of women in the Beijing region.

## Conclusion

In conclusion, the present results characterize the epidemiological characteristics of HPV infections among patients and the general population in Beijing. The HPV genotypes detected differ from the current 9-valent vaccine HPV types associated with this area. It has been hypothesized that HPV types 51, 56, 53, and 39 may be better adapted to individuals of Chinese ancestry. In the future, the development of HPV vaccines according to region may be an effective strategy.

## Data Availability

All data and materials described in manuscript are available.

## Abbreviations

CC: Cervical cancer
HPV: Human papillomavirus
HR-HPV: High-risk human papillomavirus
LR-HPV: Low-risk human papillomavirus
CCSs: Cervical cancer screening programs
